# Editorial Notes and Comments

**Published:** 1906-02

**Authors:** 


### EDITORIAL NOTES AND COMMENTS.

Our good friend, Dr. Charles S. Stockton of Newark, N. J., who has been so severely ill during the greater part of 1905, is so far recovered that he is again at his office attending his patients.

---

Dr. H. T. Stewart, Memphis Trust Bld'g, Memphis Tenn., wants a very thorough and painstaking bridge workman. He must be experienced and artistic in this line and furnish good references.

---

We wish to purchase copies of old dental books and volumes of dental journals. Write the editor stating what you have and the price.

---

Now "Sanitol" is to be "Best for the Ear" as well as "Best for the Teeth," for the composer of "Hiawatha," "Silver Heels," "Marjery," "Poppies," and other popular marches and two-steps, has named his latest and best two-step and march "Sanitol."

The music is dedicated to the president of The Sanitol Chemical Laboratory Co., Mr. Herman C. G. Luyties, and already one of the great concert bands of the country has brought to this new composition an enthusiastic reception.

We have received a copy of this march from the Sanitol Company and we understand that copies have been sent to the dentists all over the country with the compliments of the president.

The music is delightful, is full of life and has been received with great favor in musical circles. Every dentist should have one in his home and additional copies, we understand, can be secured from all music stores.

---

Ichthyol Trademark.—The Federal Tribunal of Lausanne, Switzerland, recently gave its decision in an appeal against the decision of the Court of Appeal of Berne in the action brought by the Ichthyol Co., Hamburg, proprietors of the trademark "Ichthyol," marketed in the United States by Merck & Co., of New York, to prohibit Luedy & Co., Burgdorf, from infringing the trademark. The Lausanne Court rejected the defendants' appeal and confirmed the former judgment, which ordered that the defendant firm should no longer use for their products names containing in any way the characteristic word "Ichthyol." It was proved that the trademark "Ichthyol" is the legitimate property of the Ichthyol Co., and that only this company is able to supply the sulphur preparation known under the name "Ichthyol." The defendants had pretended to supply the same preparation as supplied by the Ichthyol Co., but the Court stated that their product differed essentially in composition from the genuine article.—*Chemist and Druggist.*

